# CRISPR/Cas9 Mediated Disruption of Seminal Fluid Protein Sfp62 Induces Male Sterility in *Bombyx mori*

**DOI:** 10.3390/biology11040561

**Published:** 2022-04-07

**Authors:** Xia Xu, Jine Chen, Xin Du, Lusong Yao, Yongqiang Wang

**Affiliations:** Institute of Sericulture and Tea, Zhejiang Academy of Agricultural Sciences, Hangzhou 310021, China; xuxia@zaas.ac.cn (X.X.); chenje@zaas.ac.cn (J.C.); duxin@zaas.ac.cn (X.D.); yaols@zaas.ac.cn (L.Y.)

**Keywords:** *Sfp62*, male sterility, *Bombyx mori*, CRISPR/Cas9

## Abstract

**Simple Summary:**

In gamogenetic animals, seminal fluid proteins are essential for male fertility. In this study, we investigated the function of the seminal fluid protein Sfp62 by using the CRISPR/Cas9 system in lepidopteran model insect *Bombyx mori*. *Sfp62* mutation led to male sterility and can be inherited stably. The mutation did not affect growth and developmental nor female fertility. These data indicate that *Sfp62* is an ideal target for sterile insect technology (SIT), in which genetically modified insects are released on a large scale to mate with wild-type insects in order to reduce or even eradicate the target pests. The determining factors for the effective implementation of SIT include the strong competitiveness of the modified individuals and multi-generational effects resulting from the mutation. *Sfp62* meets these criteria and is therefore a promising target for biological pest control.

**Abstract:**

Seminal fluid proteins provide factors necessary for development, storage, and activation of sperm. Altered expression of seminal fluid proteins can lead to defect in male infertility. We investigated the function of seminal fluid protein Sfp62 in the model lepidopteran insect *Bombyx mori* using CRISPR/Cas9 mediated mutagenesis. The knockout of *BmSfp62* gene led to male sterility but has no effect on female fertility. The mutation did not affect growth and development of the silkworm of both sexes. Motility of sperm in male mutants was decreased and the mRNA expression levels of other genes encoding seminal fluid proteins were altered in *BmSfp62* mutants compared to the wild-type controls. The male sterility caused by mutation of *BmSfp62* was stably inherited. As the proteins encoded by *Sfp62* genes are conserved among lepidopteran species, *Sfp62* is a potential target for the biological management of lepidopteran pests.

## 1. Introduction

The males of gamogenetic animals produce seminal fluid proteins (SFPs) of various types that are critical for male fertility [1]. SFPs identified using transcriptome and proteome analyses include proteases, protease inhibitors, peptides, prohormones, lipases, trypsins, antioxidants, c-type lectins, and cysteine-rich secreted proteins [2,3,4,5]. SFPs provide a fluid environment for sperm and are necessary for development, storage, and activation of sperm [6,7]. Upon ejaculation, SFPs enter the genital tract of the female where these factors influence ovulation and fertilization [8,9].

In mammals, SFPs are biomarkers for male fertility [10]. About 50% of human infertility results from SFP dysfunction [11]. SFPs provide the main energy source for sperm and regulate sperm capacitation and acrosome reaction [12,13,14]. In *Mus musculus*, certain SFPs are essential for sperm–egg fusion including the membrane protein FIMP, SOF1; transmembrane protein TMEM95; the acrosome-associated protein SPACA6, DCST1, and DCST2 [15,16,17,18]. In fishes, SFPs sperm motility and fertilization are used as markers to evaluate the fish reproductive ability and to monitor sperm cryopreservation methods [19,20]. The ganglioside GM3 in SFPs is involved in the fertilization process in *Oncorhynchus mykiss* [21]. A high molecular weight glycosylated SFP is a sperm motility inhibitor in *Oreochromis niloticus* [22]. The lack of SPACA6 prevents sperm–egg fusion in *Danio rerio* [23].

In insects, SFPs not only regulate reproduction but also affect the lifespan of mating individuals and the numbers and survival rates of offspring [24,25,26,27,28,29]. In *Drosophila melanogaster*, Seminase is an essential factor for female oviposition induction, and its downregulation blocks sperm release, which in turn results in a significant reduction in post-copulation oviposition [30,31]. Acp70A (also known as sex peptide) and Acp26Aa (also known as Ovulin) regulate sperm storage and stimulate oviposition in *Drosophila* and also affect the survival of offspring [32,33,34]. In *Culex pipiens*, trypsin is essential for maintaining sperm motility and is necessary for maintenance of sperm viability in vitro [35]. In *Anopheles*, SFPs bind to symbiotic bacteria in males and transfer these bacteria to females, where they affect the female immune response [36]. In *Ceratitis capitata*, the SFPs enter the female and cause conformational changes in reproductive tract [37]. In *Teleogryllus oceanicus*, seven SFPs regulate sperm motility and enhance sperm competitiveness [38]. In *Bombyx mori* and *Plutella xylostella*, the serine protease Ser2 is an SFP necessary for male reproduction; its absence results in male sterility, but female mutants are fertile [39].

Sterile insect technology (SIT) is a new biological method for pest control [40]. The principle is that genetically modified to be sterile, are released into the field on a large scale to mate with wild-type insects to reduce or even eradicate target pests [41]. As SFPs are mainly secreted from insect gonads during the adult stage, loss of these factors does not usually impact growth and development [42]. Therefore, SFPs are potential targets for SIT [43].

In this study, we investigated the function of *Sfp62* gene in *B. mori*, a model lepidopteran insect, by CRISPR/Cas9-induced mutation of the gene. This technique has been shown to result in stable inheritance of mutations [44]. Lepidoptera is the second largest order of insects and includes more than 70% of the agriculture pests [45]. We found that the deletion of *BmSfp62* led to male sterility. Growth and development of the mutant insects was normal, and female fertility was not impacted by loss of *BmSfp62*. In females mated with male mutants, sperm motility in the bursa copulatrix was decreased. In addition, the mRNA levels of genes encoding other SFPs associated with sperm motility were altered in the *BmSfp62* mutants. Changes in SFP composition and content caused abnormalities in the fluid environment of the sperm, which presumably results in male sterility. As *Sfp62* is evolutionarily conserved among lepidopterans, the *Sfp62* is a potential target gene for lepidopteran pest control.

## 2. Materials and Methods

### 2.1. Silkworm Strains and Rearing

A multivoltine and nondiapausing silkworm strain, Nistari, was used for the experiments and raised under standard conditions [46].

### 2.2. Evolutionary Analysis

The phylogenetic relationships of Sfp62 proteins in representative lepidopteran species was inferred using neighbor-joining method [47]. The evolutionary distances were computed using Poisson correction method [48]. Phylogenetic analyses were conducted in MEGA X [49]. The alignment of Sfp62 protein sequences was created with the ClustalX2 software and GENEDOC program.

### 2.3. Quantitative Detection of Genes

TRIzol^®^ reagent (Invitrogen, Waltham, MA, USA) were applied to isolate the total RNAs. For each tissue sample, total RNAs was used with the RevertAid™ First Strand cDNA Synthesis Kit (Thermo Fisher Scientific, Waltham, MA, USA) for complementary DNA (cDNA) synthesis. Quantitative real-time PCR (qRT-PCR) analyses were performed using a SYBR Green Realtime PCR Master Mix (Thermo Fisher Scientific, USA). The PCR conditions: 95 °C for 5 min, 35 cycles at 95 °C for 15 s, and 60 °C for 1 min. The ribosomal protein 49 (*Bmrp49*) was used as an internal control. A relative quantitative method (^△△^Ct) was used to evaluate quantitative variation. The primers are listed in Table 1.

### 2.4. Plasmid Construction

Following the GGN_19_GG rule, small guide RNA (sgRNA) target sites were selected in the screened open reading frame of *BmSfp62* [50,51,52]. Two target sites of *BmSfp62* were identified. The activator was the plasmid *pBac[IE1-EGFP-Nos-Cas9]*, which encodes Cas9 under the control of the *Nos* promoter and the enhanced green fluorescent protein (EGFP) marker under control of the *IE1* promoter [53]. The effector plasmid was *pBac[IE1-DsRed2-U6-sgRNAs]*, which encodes the sgRNAs and the discosoma red fluorescent protein (DsRed) driven by *U6* and *IE1* promoters, respectively. The primers are listed in Table 1.

### 2.5. Mutant Construction

The constructed plasmids were microinjected into embryos for germline transformation. The injected eggs were incubated and raised under standard conditions. When the mutant lines reached to adults, they were arranged to mate with wild-type (WT) moths. G1 progeny were screened under a fluorescence microscope (Nikon AZ100, Tokyo, Japan). The mutant germline (Δ*BmSfp62* individuals) with DsRed (red) and EGFP (green) fluorescence were obtained by hybridization of the *Nos-Cas9* line with the *U6-sgRNA* line. Mutants with double-fluorescence were used in subsequent experiments.

### 2.6. Mutagenesis Analysis

The mutant genomic DNA was extracted by genomic DNA extraction kit (Thermo Fisher Scientific, USA). Genomic PCR was performed as follows: 94 °C for 2 min, 35 cycles of 94 °C for 15 s, 55 °C for 30 s, and 72 °C for 1 min, followed by a final extension period at 72 °C for 10 min. The PCR products were sub-cloned into pJET1.2 vectors (Thermo Fisher Scientific, USA) and sequenced. The primers are listed in Table 1.

### 2.7. Sperm Motility and Germline Transmission Assay

The bursa copulatrixes were dissected from control and Δ*BmSfp62* females at the mated adult stage. Tissues were placed in physiological saline (Sangon, Shanghai, China) on glass slides. Photos were taken with a microscope (Olympus BX51, Tokyo, Japan).

The emerging moth was set in the center of a plastic container (30 × 18 × 4.5 cm^3^). WT or Δ*BmSfp62* moth of the opposite sex was placed 10 cm from the center of the container. Mutants that mated were recorded as responsive. The response index was calculated as responsive moth number divided by total test number multiplied by 100.

### 2.8. Statistical Analysis

RT-qPCR, response index, number of eggs and progeny data were analyzed in GraphPad Prism 7. The statistically significant differences were measured by Student’s *t*-test with two-tailed distribution and error bars show ± SEM (*, *p* < 0.05; **, *p* < 0.01; ***, *p* < 0.001; n. s., *p* > 0.05). 

## 3. Results

### 3.1. Phylogenetic Identification of Sfp62

**Figure 1:**
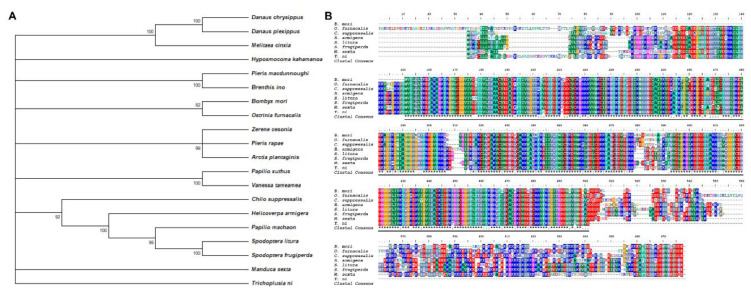
Sfp62 is highly conserved in lepidopterans. (**A**) The phylogenetic tree of Sfp62 in *B. mori* and other 19 representative lepidopteran species. GenBank accession numbers are as follows: *Bombyx mori* (XP_021204150.2), *Danaus chrysippus* (CAG9569287.1), *Danaus plexippus* (XP_032523373.1), *Melitaea cinxia* (XP_045455307.1), *Hyposmocoma kahamanoa* (XP_026322869.1), *Pieris macdunnoughi* (CAF4912374.1), *Brenthis ino* (CAH0721132.1), *Ostrinia furnacalis* (XP_028171747.1), *Zerene cesonia* (XP_038219751.1), *Pieris rapae* (XP_022123155.1), *Arctia plantaginis* (CAB3258352.1), *Papilio xuthus* (XP_013165404.1), *Vanessa tameamea* (XP_026498813.1), *Chilo suppressalis* (RVE48234.1), *Helicoverpa armigera* (PZC77022.1), *Papilio machaon* (KPJ19182.1), *Spodoptera litura* (XP_022827516.1), *Spodoptera frugiperda* (XP_035441889.1), *Manduca sexta* (XP_037295811.1), and *Trichoplusia ni* (XP_026729495.1). (**B**) Conservation of Sfp62 in *B. mori, O. furnacalis*, *C. suppressalis*, *H. armigera*, *S. litura*, *S. frugiperda*, *M. sexta*, and *T. ni*.

Sfp62 protein sequences from 20 different lepidopteran species were analyzed to evaluate phylogenetic conservation. The sequences evaluated were from *B. mori*, *Danaus chrysippus*, *Danaus plexippus*, *Melitaea cinxia*, *Hyposmocoma kahamanoa*, *Pieris macdunnoughi*, *Brenthis ino*, *Ostrinia furnacalis*, *Zerene cesonia*, *Pieris rapae*, *Arctia plantaginis*, *Papilio xuthus*, *Vanessa tameamea*, *Chilo suppressalis*, *Helicoverpa armigera*, *Papilio machaon*, *Spodoptera litura*, *Spodoptera frugiperda*, *Manduca sexta*, and *Trichoplusia ni*. Phylogenetic analysis of Sfp62 protein sequences indicated that the protein is highly conserved (Figure 1A). In addition, we aligned the Sfp62 sequences of six pest species and silkworm (Figure 1B). Sfp2 protein sequence from *B. mori* had 87.11% identity with *O. furnacalis*, 83.92% with *C. suppressalis*, 85.01% with *H. armigera*, 63.99% with *S. litura*, 85.76% with *S. frugiperda*, 85.51% with *M. sexta*, and 84.39% with *T. ni* (Figure 1B). These data indicate that findings in *B. mori* regarding the function of Sfp2 are likely applicable to other lepidopterans.

### 3.2. Expression Pattern of BmSfp62

**Figure 2:**
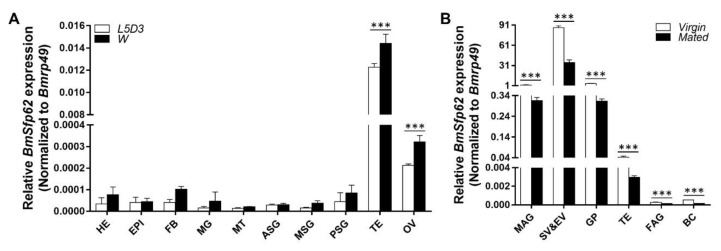
*BmSfp62* is highly expressed in testis. (**A**) *BmSfp62* expression in 10 tissues: head (HE), epidermis (EPI), fat body (FB), midgut (MG), Malpighian tubules (MT), anterior silk gland (ASG), middle silk gland (MSG), posterior silk gland (PSG), testis (TE) and ovary (OV) at L5D3 and W stages. (**B**) *BmSfp62* expression in male accessory gland (MAG), seminal vesicle and ejaculatory vesicle (SV&EV), glandula prostatica (GP), testis (TE), female accessory gland (FAG), and bursa copulatrix (BC) in virgin and mated adults. *BmSfp62* mRNA expression level was normalized to expression of *Bmrp49*. The data shown are means ± S.E.M. (*n* = 3). Asterisks indicate significant differences with a two-tailed *t*-test: *** *p* < 0.001.

The testis of silkworm in adult stage were nearly spherical rather than renal in the larval stage. Male gradually matured from the fifth larval instar to the adult stage. Therefore, we tested the mRNA expression of *BmSfp62* in each representative stages. The expressions were first analyzed at day 3 of the fifth instar larvae (L5D3) and the wandering stage (W). In individuals from both developmental stages, *BmSfp62* mRNA was much more highly expressed in the testis than head, epidermis, fat body, midgut, Malpighian tubules, anterior silk gland, middle silk gland, posterior silk gland, and ovary (Figure 2A). We then quantified the expression in adult tissues. *BmSfp62* mRNA was more highly expressed in the seminal vesicle and ejaculatory vesicle than male accessory gland, glandula prostatica, testis, female accessory gland, and bursa copulatrix in both virgin and mated adults (Figure 2B). In the silkworm gonads, the mRNA expression of *BmSfp62* increased significantly with development. These results suggest that the Sfp62 is important in male fertility.

### 3.3. CRISPR/Cas9-Mediated BmSfp62 Mutation Leads to Male Sterility

**Figure 3:**
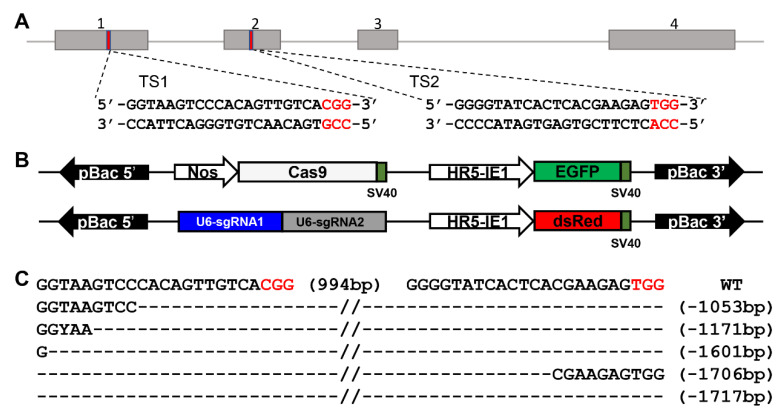
*BmSfp62* knockout using CRISPR/Cas9. (**A**) Genomic structure of *BmSfp62*. (**B**) Schematic representations the *Nos-Cas9* and *U6-sgRNA*. (**C**) Base deletion. Black is target sequences; red is PAM sequences. The deletion size is indicated to the right of the sequence.

Two target sites were selected in exons of the *BmSfp62* gene; the fragment containing the two sites was 994 bp (Figure 3A). The activator line vector *pBac[IE1-EGFP-Nos-Cas9]* is designed to express the fluorescent marker EGFP and the effector line *pBac[IE1-DsRed-U6-sgRNAs]* is designed to express the fluorescent marker DsRed (Figure 3B). Genomic DNA was extracted from the mutants with EGFP and DsRed fluorescent markers to characterize deletions between the two target sites. Different numbers of bases were deleted in different mutant individuals (Figure 3C).

**Figure 4:**
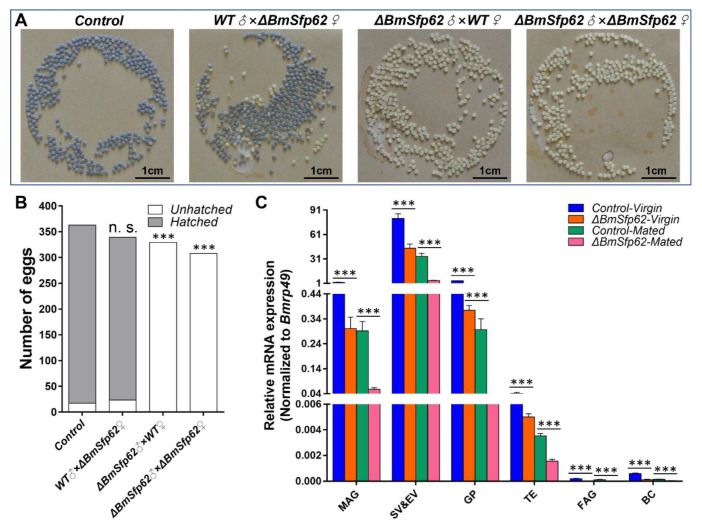
Deletion of *BmSfp62* results in male sterility. (**A**) Eggs produced by crosses. (**B**) Eggs hatched and unhatched. (**C**) Relative mRNA expression of *BmSfp62* in reproductive adult tissues. Virgin WT is in blue, virgin Δ*BmSfp62* is in orange, mated WT is in green, and mated Δ*BmSfp62* is in pink. *BmSfp62* mRNA expression level was normalized to *Bmrp49*. The data shown are means ± S.E.M. (*n* = 3). Asterisks indicate significant differences with a two-tailed *t*-test: *** *p* < 0.001; n. s. *p* > 0.05.

We then mated WT males with both Δ*BmSfp62* and WT females and Δ*BmSfp62* males with both WT and Δ*BmSfp62* females and examined numbers of eggs produced (Figure 4A). All crosses resulted in similar numbers of eggs laid; however, eggs produced by females mated with Δ*BmSfp62* males did not hatch. The control WT cross produced 363 ± 10 eggs; WT males mated with Δ*BmSfp62* females produced 339 ± 10 eggs; Δ*BmSfp62* males mated with WT females produced 329 ± 10 eggs; Δ*BmSfp62* males mated with Δ*BmSfp62* females produced 308 ± 10 eggs (*n* = 30/group). Almost all control eggs hatched (~95%, 346/363) as did eggs of WT males mated with Δ*BmSfp62* females (~93%, 316/339), but no eggs of Δ*BmSfp62* males mated with WT or Δ*BmSfp62* females hatched (~0%, 0/329; ~0%, 0/308, respectively) (Figure 4B). Subsequently, we tested the mRNA expression of *BmSfp62* in adult tissues. *BmSfp62* mRNA expression was significantly downregulated in Δ*BmSfp62* individuals compared with WT individuals (Figure 4C).

### 3.4. ΔBmSfp62 Sperm Motility Is Decreased and SPF Expression Is Altered

We next evaluated sperm motility in the bursa copulatrix of WT females after mating with WT or Δ*BmSfp62* males. Normally, eupyrene sperm do not move on their own but are carried by the movement of apyrene sperm. Apyrene sperm motility was significantly decreased in the mutant compared to WT controls (Videos S1 and S2).

**Figure 5:**
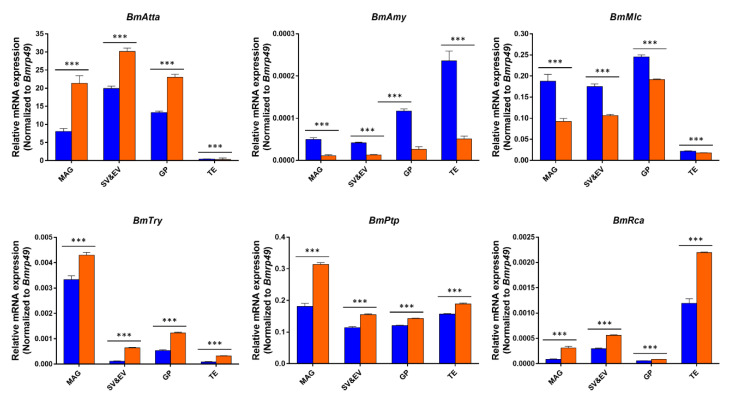
Expression of genes associated with sperm development and motility is altered in Δ*BmSfp62* individuals. mRNA levels of *BmAtta* (GU244351.1), *BmAmy* (NM_001173153.1), *BmMlc* (XM_012694217.1), *BmTry* (XM_012688545.2), *BmPtp* (NM_001047017.1), and *BmRca* (XM_004930660.3). WT is in blue, Δ*BmSfp62* is in orange. mRNA expression level was normalized to *Bmrp49*. The data shown are means ± S.E.M. (*n* = 3). Asterisks indicate significant differences with a two-tailed *t*-test: *** *p* < 0.001.

The mRNA levels of genes encoding sperm motility-related SFPs *BmAtta*, *BmTry*, *BmPtp*, and *BmRca* were significantly up-regulated in virgin adult gonads Δ*BmSfp62* individuals compared to WT, whereas *BmAmy* and *BmMlc* were significantly downregulated in the mutant (Figure 5). Thus, deletion of *BmSfp62* leads to the altered expression of other genes encoding SFPs that are essential for fertility.

### 3.5. Mutation of BmSfp62 Does Not Affect Adult Competitiveness and Is Stably Inherited

**Figure 6:**
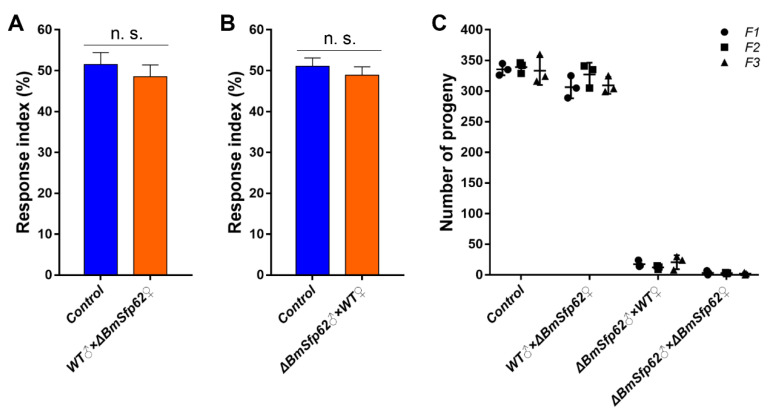
The *BmSfp62* mutation does not impair competitiveness and is inherited. (**A**) Response indices of WT and Δ*BmSfp62* females. (**B**) Response indices of WT and Δ*BmSfp62* males. (**C**) Number of progenies with indicated mutations in F1 (circles), F2 (squares), and F3 (triangles) generations. The data shown are means ± S.E.M. (*n* = 30/group). N.S. indicates no significance by a two-tailed *t*-test. Horizontal bars indicate means (*n* = 3).

We used the percentage of successful mating as a response index to evaluate the adult competitiveness of the mutant females and males. There was no significant difference in competitiveness of WT and Δ*BmSfp62* females (51.52% and 48.48%, respectively; *n* = 30/group) nor was there any significant difference in competitiveness of WT and Δ*BmSfp62* males (51.09% and 48.91%, respectively; *n* = 30/group) (Figure 6A, B). Heritability stability was analyzed by hybridization of the *Nos-Cas9* and *U6-sgRNAs* lines. In successive generations, Δ*BmSfp62* male sterility was observed (Figure 6C). Thus, the *BmSfp62* mutation did not affect competitiveness, and the resulting male sterility was stably inherited.

## 4. Discussion

Seminal fluid proteins are essential for male reproduction. There are many SFPs, and their characteristics and functions vary [54]. Here, we conducted a functional analysis of *Sfp62* using the silkworm as a model. At the protein level, Sfp62 is highly conserved across lepidopteran species. *BmSfp62* is highly expressed in male gonads; it was detected in other tissues but at low levels. We evaluated the physiological function of *BmSfp62* gene in male reproduction by CRISPR/Cas9 technology. The technology enables accurate and efficient targeting of candidate genes [55,56]. The *BmSfp62* mutation caused male sterility but did not decrease female fertility. Growth and reproductive behaviors of mutants were normal, but the sperm motility of the male *BmSfp62* mutant was dramatically decreased compared to wild-type sperm, resulting in fertilization failure. Females mated with *BmSfp62* mutant males laid normal numbers of eggs but did not hatch.

Mutation of *BmSfp62* led to loss of *BmSfp62* and alters expression of other genes that encode seminal fluid proteins including *BmAtta*, *BmAmy*, *BmMlc*, *BmTry*, *BmPtp*, and *BmRca*. In *D. melanogaster*, *Atta*, an immune defense gene, is highly expressed in virgin adults and regulates sperm competitiveness [57]. In *D. melanogaster*, *Amy* is expressed in the spermatid and mature sperm; the protein it encodes binds cAMP-dependent kinase to regulate spermatogenesis and sperm capacitation in human and mouse [58]. The *Mlc* gene encodes a motor protein that is required for sperm storage and release in *D. melanogaster* [59,60]. The hydrolase encoded by *Try* regulates the hydrolyzation of sex peptides that influence sperm motility and reproduction [61]. *Ptp* is expressed in lepidopteran testis, and its homolog in *D. melanogaster* is critical for efficient ATP synthesis necessary for sperm motility [62]. The SFP encoded by *Rca* regulates sperm quality and male fertility by influencing Ca^+^ levels in seminal fluid in *Rattus norvegicus* [63]. The abnormal expression of SPFs resulting from *BmSfp62* deficiency may contribute to the observed male sterility. Sperm motility and the levels of SFPs in the female’s bursa copulatrix after mating influence the efficiency of sperm–egg fusion [64,65]. Loss of *BmSfp62* gene function led to a decrease in sperm motility, which likely also impaired fertility.

In summary, our study demonstrated that the mutation of *Sfp62* led to stably inherited male sterility by CRISPR/Cas9 system in *B. mori*. The *BmSfp62* gene deletion caused male sterility, but females were fertile, and the mutation did not affect other growth and reproduction indicators. *BmSfp62* mutant females released into the environment will mate with WT males and pass on effectively. The next generation of mutant males will be sterile but able to mate. Mating with mutants should eventually suppress the pest population. Further, *BmSfp62* is evolutionarily conserved among lepidopterans, making it an ideal target for SIT-mediated control of lepidopteran pests.

## 5. Conclusions

We investigated the function of *BmSfp62* in the lepidopteran model insect, *B. mori*. The *BmSfp62* mutant males were sterile, but females were fertile. The mutation was stably inherited and did not affect other growth and reproductive indicators. *BmSfp62* deletion decreased sperm motility and altered mRNA expression levels of other genes encoding seminal fluid proteins. Due to it evolutionary conservation in lepidopterans, *BmSfp62* is a possible target for biological control of lepidopteran pests.

## Figures and Tables

**Table 1:** Primers used in detection.

Primer Name	Primer Sequence (5′-3′)
*qRT-PCR analysis*	
BmSfp62-F	GGCCCTTCCTATACACGTCA
BmSfp62-R	CGTCACTATCGTTTGCAGCA
BmAtta-F	GCGGACTGGACTACATGTTC
BmAtta-R	TCAAACTTCTTGAAGCCGGC
BmAmy-F	CATGGGTGTTGCTGGTTTCA
BmAmy-R	TGCGACTGATAGCTTCACCA
BmMlc-F	TCCCATCTACAGCCAAGCAA
BmMlc-R	TAGGGGATCATGCCGTCATC
BmTry-F	CGTACCGACTTTCAGGAGGA
BmTry-R	CAGATGATGTGGTGCCTTCG
BmPtp-F	CGCCGAGAAGTACAAGAACG
BmPtp-R	TACGGTAAGTGTAGGCGGTC
BmRca-F	ATGACCATAGACACGGACGG
BmRca-R	TTCATAGAGGCGGAGGTCAC
BmRP49-F	TCAATCGGATCGCTATGACA
BmRP49-R	ATGACGGGTCTTCTTGTTGG
*Plasmid construction*	
U6-F	CTCACTATAGGGCGAATTGGAGGTTATGTAGTACACATTGTTGTA
U6-R	TTTTCTTGTTATAGATATCAAAAAAAGCACCGACTCGGTG
Overlap-F	GCTAGCCATTGACTCCGCGGAGGTTATGTAGTACACATTGTTGTA
Overlap-R	CCGCGGAGTCAATGGCTAGCAAAAAAGCACCGACTCGGTG
Sg1-F	GGTAAGTCCCACAGTTGTCAGTTTTAGAGCTAGAAATAGCAAGTT
Sg1-R	TGACAACTGTGGGACTTACCACTTGTAGAGCACGATATTTTGTAT
Sg2-F	GGGGTATCACTCACGAAGAGGTTTTAGAGCTAGAAATAGCAAGTT
Sg2-R	CTCTTCGTGAGTGATACCCCACTTGTAGAGCACGATATTTTGTAT
*Identification of mutations*	
TS-F	ATGCAGAATGACCACGGTGGG
TS-R	CGTCGAGCCCTTCGTCTCAA

## Data Availability

The data presented in this study are available on request from the corresponding author.

## Supplementary Materials

The following supporting information can be downloaded at: https://www.mdpi.com/article/10.3390/biology11040561/s1, Video S1. Sperm motility in bursa copulatrix of WT female mated with WT male; Video S2. Sperm motility in bursa copulatrix of WT female mated with Δ*BmSfp62* male.

## References

[1] Davidovics R., Saw Y.L., Brown C.O., Prinz M., McKiernan H.E., Danielson P.B., Legg K.M. (2022). High-throughput seminal fluid identification by automated immunoaffinity mass spectrometry. J. Forensic. Sci..

[2] Laflamme B.A., Wolfner M.F. (2013). Identification and function of proteolysis regulators in seminal fluid. Mol. Reprod. Dev..

[3] Findlay G.D., Sitnik J.L., Wang W., Aquadro C.F., Clark N.L., Wolfner M.F. (2014). Evolutionary rate covariation identifies new members of a protein network required for Drosophila melanogaster female post-mating responses. PLoS Genet..

[4] Ram K.R., Wolfner M.F. (2007). Sustained post-mating response in Drosophila melanogaster requires multiple seminal fluid proteins. PLoS Genet..

[5] Sitaram N., Nagaraj R. (1995). Seminal plasmin. Bioessays.

[6] Amaro I.A., Ahmed-Braimah Y.H., League G.P., Pitcher S.A., Avila F.W., Cruz P.C., Harrington L.C., Wolfner M.F. (2021). Seminal fluid proteins induce transcriptome changes in the Aedes aegypti female lower reproductive tract. BMC Genom..

[7] Ramm S.A. (2020). Seminal fluid and accessory male investment in sperm competition. Philos. Trans. R. Soc. Lond. B Biol. Sci..

[8] Sirot L.K. (2019). On the evolutionary origins of insect seminal fluid proteins. Gen. Comp. Endocrinol..

[9] Robertson S.A. (2007). Seminal fluid signaling in the female reproductive tract: Lessons from rodents and pigs. J. Anim. Sci..

[10] Druart X., de Graaf S. (2018). Seminal plasma proteomes and sperm fertility. Anim. Reprod. Sci..

[11] Minhas S., Bettocchi C., Boeri L., Capogrosso P., Carvalho J., Cilesiz N.C., Cocci A., Corona G., Dimitropoulos K., Gul M. (2021). European Association of Urology Guidelines on Male Sexual and Reproductive Health: 2021 Update on Male Infertility. Eur. Urol..

[12] Brown C.O., Robbins B.L., McKiernan H.E., Danielson P.B., Legg K.M. (2021). Direct seminal fluid identification by protease-free high-resolution mass spectrometry. J. Forensic. Sci..

[13] Rodriguez-Martinez H., Kvist U., Ernerudh J., Sanz L., Calvete J.J. (2011). Seminal plasma proteins: What role do they play?. Am. J. Reprod. Immunol..

[14] Costello S., Michelangeli F., Nash K., Lefievre L., Morris J., Machado-Oliveira G., Barratt C., Kirkman-Brown J., Publicover S. (2009). Ca^2+^-stores in sperm: Their identities and functions. Reproduction.

[15] Altschul S.F., Madden T.L., Schaffer A.A., Zhang J., Zhang Z., Miller W., Lipman D.J. (1997). Gapped BLAST and PSI-BLAST: A new generation of protein database search programs. Nucleic. Acids Res..

[16] Lamas-Toranzo I., Hamze J.G., Bianchi E., Fernandez-Fuertes B., Perez-Cerezales S., Laguna-Barraza R., Fernandez-Gonzalez R., Lonergan P., Gutierrez-Adan A., Wright G.J. (2020). TMEM95 is a sperm membrane protein essential for mammalian fertilization. Elife.

[17] Noda T., Lu Y., Fujihara Y., Oura S., Koyano T., Kobayashi S., Matzuk M.M., Ikawa M. (2020). Sperm proteins SOF1, TMEM95, and SPACA6 are required for sperm-oocyte fusion in mice. Proc. Natl. Acad. Sci. USA.

[18] Deneke V.E., Pauli A. (2021). The Fertilization Enigma: How Sperm and Egg Fuse. Annu. Rev. Cell Dev. Biol..

[19] Loir M., Labbe C., Maisse G., Pinson A., Boulard G., Mourot B., Chambeyron F. (1990). Proteins of seminal fluid and spermatozoa in the trout (Oncorhynchus mykiss): Partial characterization and variations. Fish Physiol. Biochem..

[20] Rasmussen T.H., Korsgaard B. (2004). Estrogenic octylphenol affects seminal fluid production and its biochemical composition of eelpout (*Zoarces viviparus*). Comp. Biochem. Physiol. Part C Toxicol. Pharmacol..

[21] Yu S., Kojima N., Hakomori S.I., Kudo S., Inoue S., Inoue Y. (2002). Binding of rainbow trout sperm to egg is mediated by strong carbohydrate-to-carbohydrate interaction between (KDN)GM3 (deaminated neuraminyl ganglioside) and Gg3-like epitope. Proc. Natl. Acad. Sci. USA.

[22] Mochida K., Kondo T., Matsubara T., Adachi S., Yamauchi K. (1999). A high molecular weight glycoprotein in seminal plasma is a sperm immobilizing factor in the teleost Nile tilapia, Oreochromis niloticus. Dev. Growth Differ..

[23] Binner M.I., Kogan A., Panser K., Schleiffer A., Deneke V.E., Pauli A. (2021). The Sperm Protein Spaca6 is Essential for Fertilization in Zebrafish. Front. Cell Dev. Biol..

[24] Koppik M., Fricke C. (2017). Gene expression changes in male accessory glands during ageing are accompanied by reproductive decline in Drosophila melanogaster. Mol. Ecol..

[25] Carmel I., Tram U., Heifetz Y. (2016). Mating induces developmental changes in the insect female reproductive tract. Curr. Opin. Insect Sci..

[26] Simmons L.W., Beveridge M., Li L., Tan Y.F., Millar A.H. (2014). Ontogenetic changes in seminal fluid gene expression and the protein composition of cricket seminal fluid. Evol. Dev..

[27] Gasparini C., Marino I.A., Boschetto C., Pilastro A. (2010). Effect of male age on sperm traits and sperm competition success in the guppy (Poecilia reticulata). J. Evol. Biol..

[28] Sirot L.K., Buehner N.A., Fiumera A.C., Wolfner M.F. (2009). Seminal fluid protein depletion and replenishment in the fruit fly, Drosophila melanogaster: An ELISA-based method for tracking individual ejaculates. Behav. Ecol. Sociobiol..

[29] Avila F.W., Sirot L.K., LaFlamme B.A., Rubinstein C.D., Wolfner M.F. (2011). Insect seminal fluid proteins: Identification and function. Annu. Rev. Entomol..

[30] Ignotz G.G., Cho M.Y., Suarez S.S. (2007). Annexins are candidate oviductal receptors for bovine sperm surface proteins and thus may serve to hold bovine sperm in the oviductal reservoir. Biol. Reprod..

[31] LaFlamme B.A., Ram K.R., Wolfner M.F. (2012). The Drosophila melanogaster seminal fluid protease “seminase” regulates proteolytic and post-mating reproductive processes. PLoS Genet..

[32] Ben Chehida Y., Denis B., Claisse G., Joly D. (2014). What the study of seminal fluid proteins in Drosophila tells us about the evolution of reproduction. Med. Sci..

[33] McGeary M.K., Findlay G.D. (2020). Molecular evolution of the sex peptide network in Drosophila. J. Evol. Biol..

[34] Kubli E. (2003). Sex-peptides: Seminal peptides of the Drosophila male. Cell Mol. Life Sci..

[35] Stephens K., Cardullo R.A., Thaler C.D. (2018). Culex pipiens sperm motility is initiated by a trypsin-like protease from male accessory glands. Mol. Reprod. Dev..

[36] Wang S., Dos-Santos A.L.A., Huang W., Liu K.C., Oshaghi M.A., Wei G., Agre P., Jacobs-Lorena M. (2017). Driving mosquito refractoriness to Plasmodium falciparum with engineered symbiotic bacteria. Science.

[37] Scolari F., Gomulski L.M., Ribeiro J.M., Siciliano P., Meraldi A., Falchetto M., Bonomi A., Manni M., Gabrieli P., Malovini A. (2012). Transcriptional profiles of mating-responsive genes from testes and male accessory glands of the Mediterranean fruit fly, Ceratitis capitata. PLoS ONE.

[38] Simmons L.W., Lovegrove M. (2017). Socially cued seminal fluid gene expression mediates responses in ejaculate quality to sperm competition risk. Proc. Biol. Sci..

[39] Xu X., Wang Y., Bi H., Xu J., Liu Z., Niu C., He L., James A.A., Li K., Huang Y. (2020). Mutation of the seminal protease gene, serine protease 2, results in male sterility in diverse lepidopterans. Insect Biochem. Mol. Biol..

[40] De Castro Poncio L., Dos Anjos F.A., de Oliveira D.A., Rebechi D., de Oliveira R.N., Chitolina R.F., Fermino M.L., Bernardes L.G., Guimaraes D., Lemos P.A. (2021). Novel Sterile Insect Technology Program Results in Suppression of a Field Mosquito Population and Subsequently to Reduced Incidence of Dengue. J. Infect. Dis..

[41] Marec F., Vreysen M.J.B. (2019). Advances and Challenges of Using the Sterile Insect Technique for the Management of Pest Lepidoptera. Insects.

[42] Bieniek J.M., Drabovich A.P., Lo K.C. (2016). Seminal biomarkers for the evaluation of male infertility. Asian J. Androl..

[43] Benedict M.Q. (2021). Sterile Insect Technique: Lessons From the Past. J. Med. Entomol..

[44] Komor A.C., Badran A.H., Liu D.R. (2017). CRISPR-Based Technologies for the Manipulation of Eukaryotic Genomes. Cell.

[45] Roscoe L.E., Silk P., Eveleigh E.S. (2016). Evidence of Male Hair Pencil Pheromone in *Choristoneura fumiferana* (Lepidoptera: Tortricidae). J. Insect Sci..

[46] Tan A., Tanaka H., Tamura T., Shiotsuki T. (2005). Precocious metamorphosis in transgenic silkworms overexpressing juvenile hormone esterase. Proc. Natl. Acad. Sci. USA.

[47] Saitou N., Nei M. (1987). The neighbor-joining method: A new method for reconstructing phylogenetic trees. Mol. Biol. Evol..

[48] Zuckerkandl E. (1965). Evolutionary divergence and convergence in proteins. Evolving Genes & Proteins.

[49] Kumar S., Stecher G., Li M., Knyaz C., Tamura K. (2018). MEGA X: Molecular Evolutionary Genetics Analysis across Computing Platforms. Mol. Biol. Evol..

[50] Hwang W.Y., Fu Y., Reyon D., Maeder M.L., Tsai S.Q., Sander J.D., Peterson R.T., Yeh J.R., Joung J.K. (2013). Efficient genome editing in zebrafish using a CRISPR-Cas system. Nat. Biotechnol..

[51] Doudna J.A., Charpentier E. (2014). Genome editing. The new frontier of genome engineering with CRISPR-Cas9. Science.

[52] Hsu P.D., Lander E.S., Zhang F. (2014). Development and applications of CRISPR-Cas9 for genome engineering. Cell.

[53] Xu J., Chen R.M., Chen S.Q., Chen K., Tang L.M., Yang D.H., Yang X., Zhang Y., Song H.S., Huang Y.P. (2018). Identification of a germline-expression promoter for genome editing in Bombyx mori. Insect Sci..

[54] Rodriguez-Martinez H., Martinez E.A., Calvete J.J., Pena Vega F.J., Roca J. (2021). Seminal Plasma: Relevant for Fertility?. Int. J. Mol. Sci..

[55] Ma Y.W., Zhang L.F., Huang X.X. (2014). Genome modification by CRISPR/Cas9. FEBS J..

[56] Manghwar H., Lindsey K., Zhang X., Jin S. (2019). CRISPR/Cas System: Recent Advances and Future Prospects for Genome Editing. Trends. Plant. Sci..

[57] McGraw L.A., Gibson G., Clark A.G., Wolfner M.F. (2004). Genes regulated by mating, sperm, or seminal proteins in mated female Drosophila melanogaster. Curr. Biol..

[58] Furusawa M., Ohnishi T., Taira T., Iguchi-Ariga S.M., Ariga H. (2001). AMY-1, a c-Myc-binding protein, is localized in the mitochondria of sperm by association with S-AKAP84, an anchor protein of cAMP-dependent protein kinase. J. Biol. Chem..

[59] McGraw L.A., Clark A.G., Wolfner M.F. (2008). Post-mating gene expression profiles of female Drosophila melanogaster in response to time and to four male accessory gland proteins. Genetics.

[60] Chakravorty S., Vu H., Foelber V., Vigoreaux J.O. (2014). Mutations of the Drosophila myosin regulatory light chain affect courtship song and reduce reproductive success. PLoS ONE.

[61] Sitnik J.L., Gligorov D., Maeda R.K., Karch F., Wolfner M.F. (2016). The Female Post-Mating Response Requires Genes Expressed in the Secondary Cells of the Male Accessory Gland in Drosophila melanogaster. Genetics.

[62] Sugahara R., Jouraku A., Nakakura T., Minaba M., Yamamoto T., Shinohara Y., Miyoshi H., Shiotsuki T. (2017). Tissue-specific expression and silencing phenotypes of mitochondrial phosphate carrier paralogues in several insect species. Insect Mol. Biol..

[63] Silva A.M.S., Socorro S., Hurtado de Llera A., Vaz C.V., Correia S., Maia C.J. (2020). Overexpression of regucalcin mitigates the ageing-related changes in oxidative stress and sperm quality. Theriogenology.

[64] Primakoff P., Myles D.G. (2002). Penetration, adhesion, and fusion in mammalian sperm-egg interaction. Science.

[65] Scolari F., Khamis F.M., Perez-Staples D. (2021). Beyond Sperm and Male Accessory Gland Proteins: Exploring Insect Reproductive Metabolomes. Front. Physiol..

